# Supplementary figures and images for: Evaluation of the severity of nonalcoholic fatty liver disease through analysis of serum exosomal miRNA expression
Source: PLoS One. 2021 Aug 6;16(8):e0255822. doi: 10.1371/journal.pone.0255822 (PMC8345824; doi:10.1371/journal.pone.0255822)

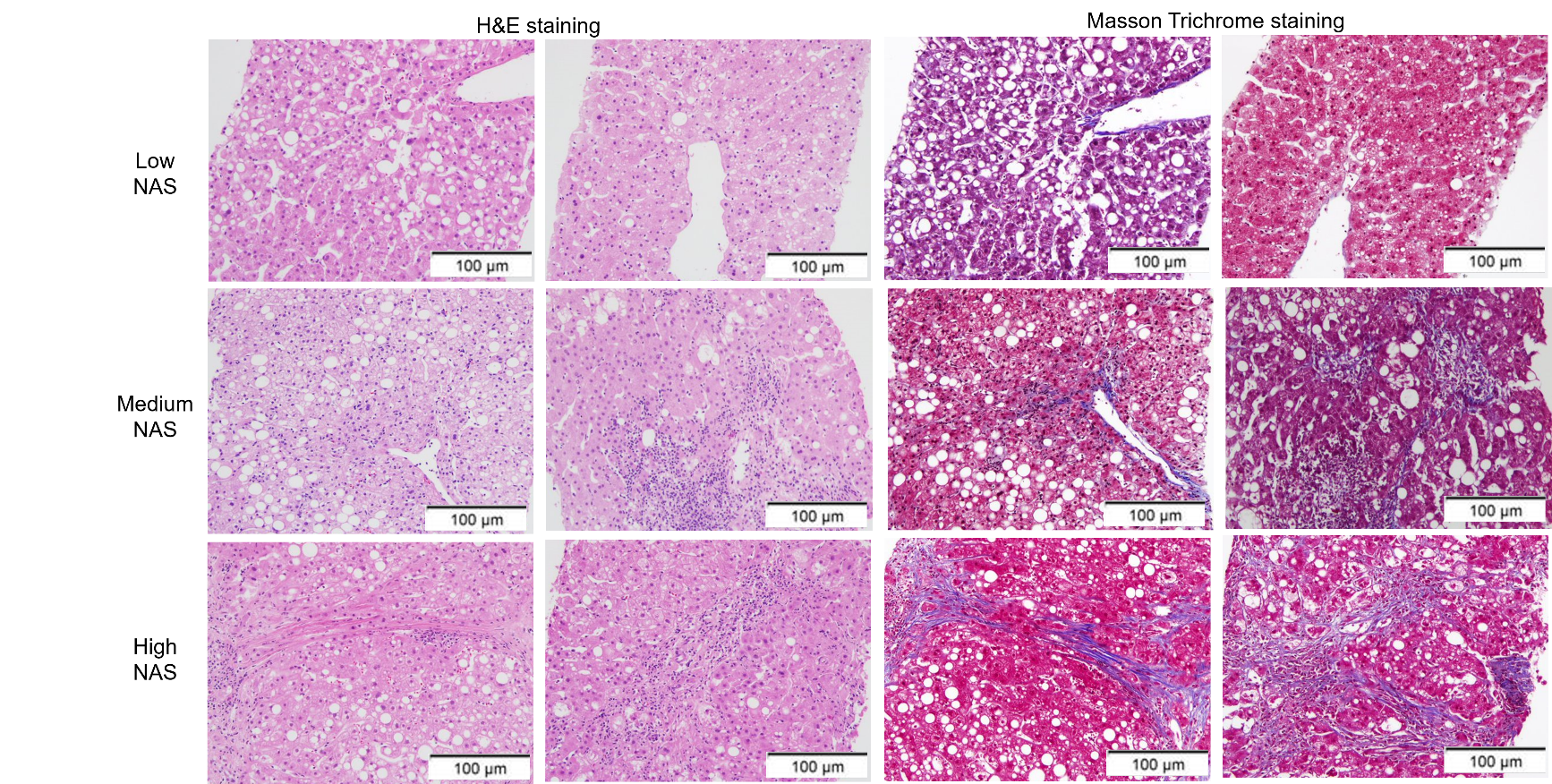

Supplement: S1 Fig — Steatosis scores: 0 (< 5%), 1 (5%–33%), 2 (> 33%–66%), or 3 (> 66%). Inflammation scores: 0 (no foci), 1 (< 2 foci per 200× field), 2 (2–4 foci per 200× field), or 3 (> 4 foci per 200× field). Hepatocyte ballooning scores: 0 (none), 1 (few), or 2 (many). Fibrosis stage: 0 (none), 1 (perisinusoidal or periportal), 2 (perisinusoidal and portal/periportal), 3 (bridging fibrosis), or 4 (cirrhosis). *NAS, nonalcoholic fatty liver disease (NAFLD) activity score; H&E, hematoxylin and eosin staining. (TIF) [file pone.0255822.s001.tif]

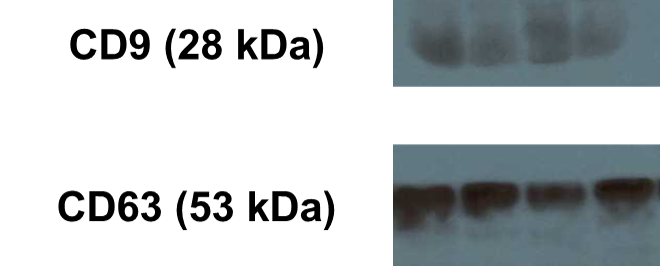

Supplement: S2 Fig — Expression levels of CD9 (28 kDa) and CD63 (53 kDa) in four samples. (TIF) [file pone.0255822.s002.tif]

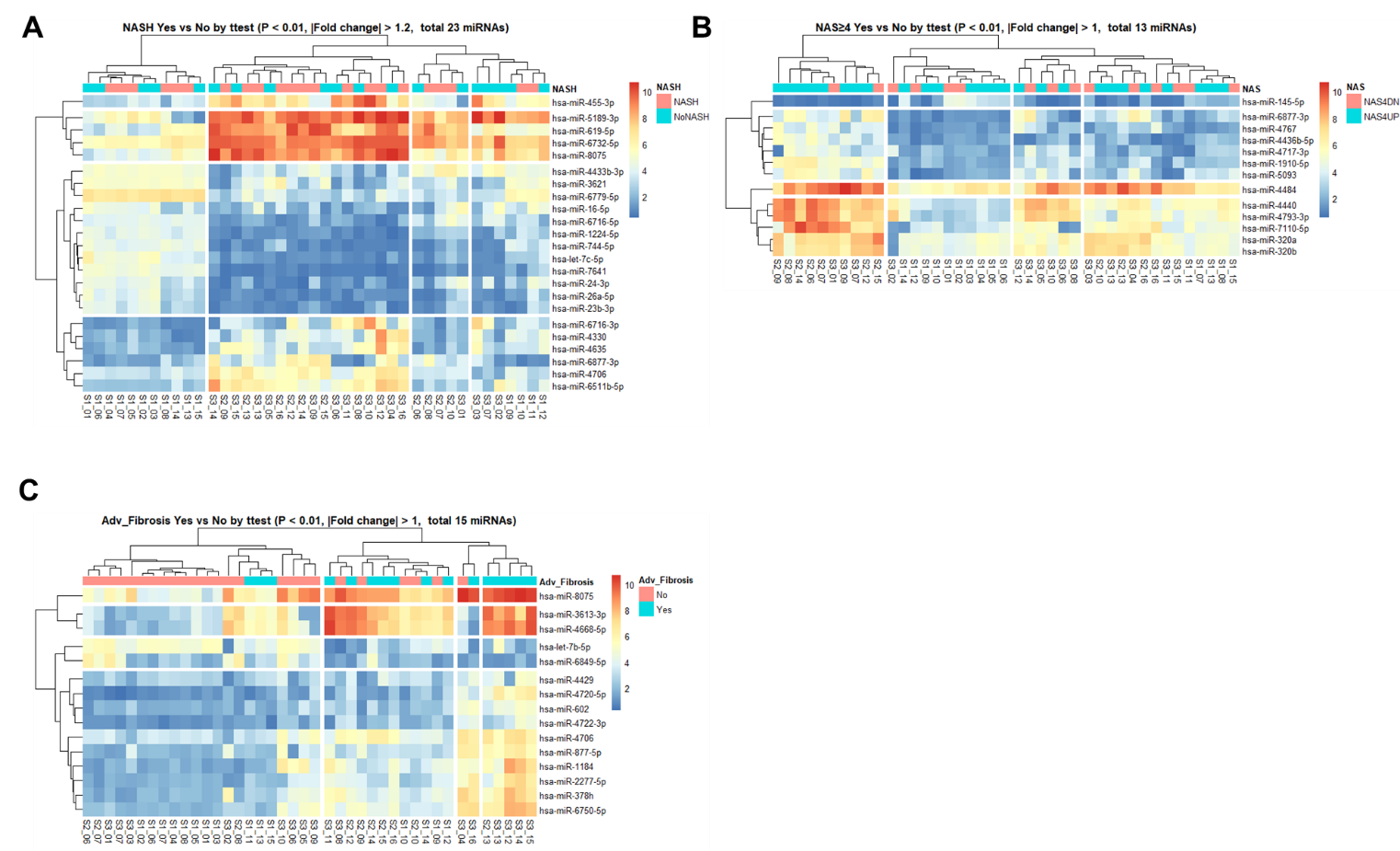

Supplement: S3 Fig — Heatmaps of differentially expressed miRNAs (rows) from 41 patients (columns) are displayed for NASH (A), NAS (B), and fibrosis (C). The classification of the variable for each patient at the top of the rows is displayed in the bar. Each row indicates the miRNAs identified by a t-test, and each column indicates a patient. Each row and column pair was clustered by the k-means clustering method using the package “pheatmap” in R, and divided into four sections. NASH, nonalcoholic steatohepatitis; NAS, nonalcoholic fatty liver disease (NAFLD) activity score. (TIF) [file pone.0255822.s003.tif]

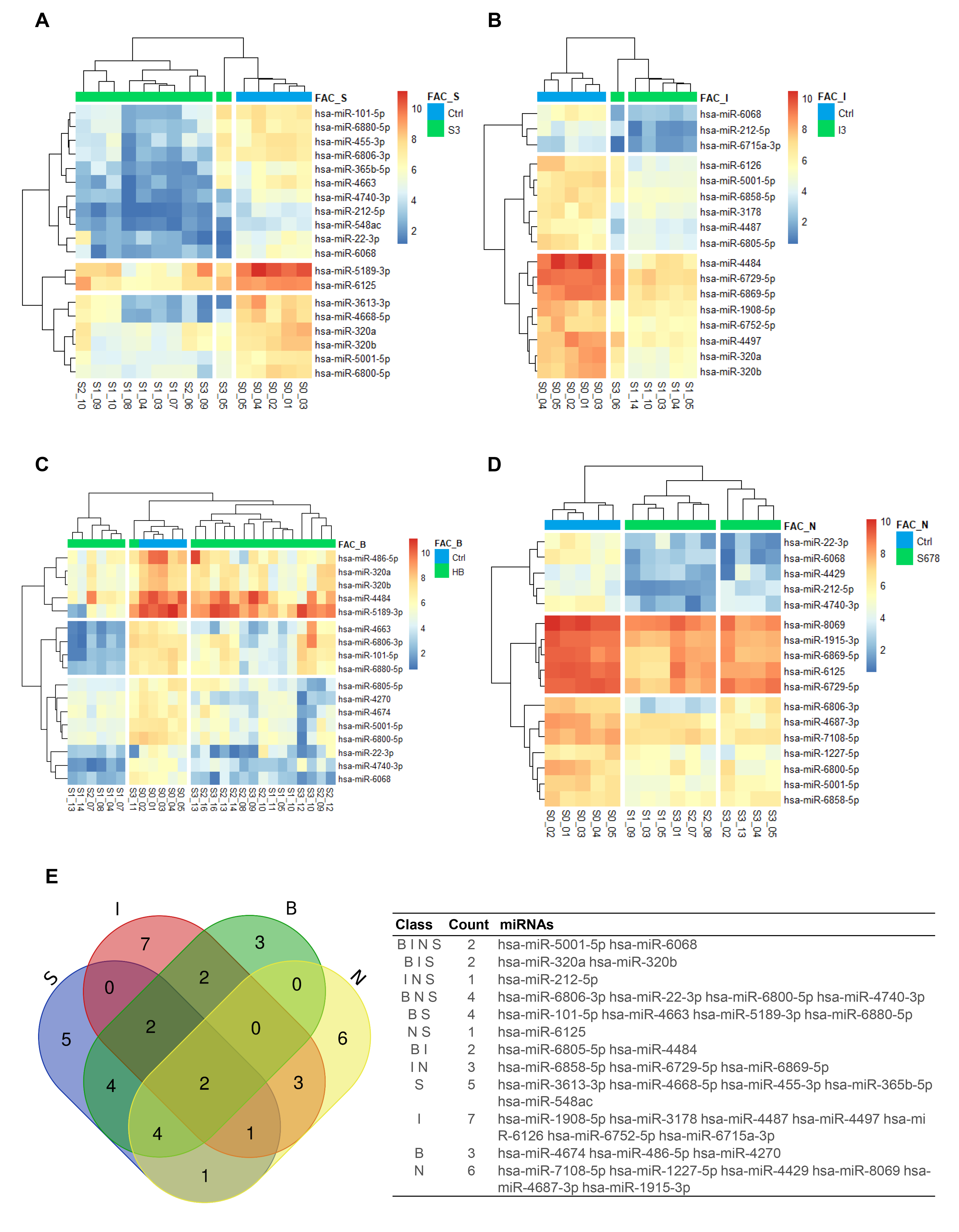

Supplement: S4 Fig — The highest score among the four scores (steatosis score 3; n = 10, inflammation score 3; n = 6, ballooning score 1 or 2; n = 22, and NAS 6, 7, or 8; n = 10) and five normal controls were compared. (A) A total of 19 miRNAs with a difference in expression between five normal controls and 10 steatosis score 3 (S3; p < 0.001 and |fold change| > 1.8). (B) A total of 17 miRNAs with a difference in expression between five normal controls and six inflammation score 3 (I3; p < 0.001 and |fold change| > 1.5). (C) A total of 17 miRNAs with a difference in expression between five normal controls and 22 ballooning scores of 1 or 2 (p < 0.001 and |fold change| > 1.8). HB indicates a high ballooning score, considered as a score of 1 or 2. (D) A total of 17 miRNAs with a difference in expression between five normal controls and 10 NAS scores of 6, 7, or 8 (S678; p < 0.001 and |fold change| > 1.1). (E) Venn diagram and table by variable for each miRNA. S, I, B, and N indicate steatosis, inflammation, ballooning, and NAS (nonalcoholic fatty liver disease (NAFLD) activity score), respectively. (TIF) [file pone.0255822.s004.tif]

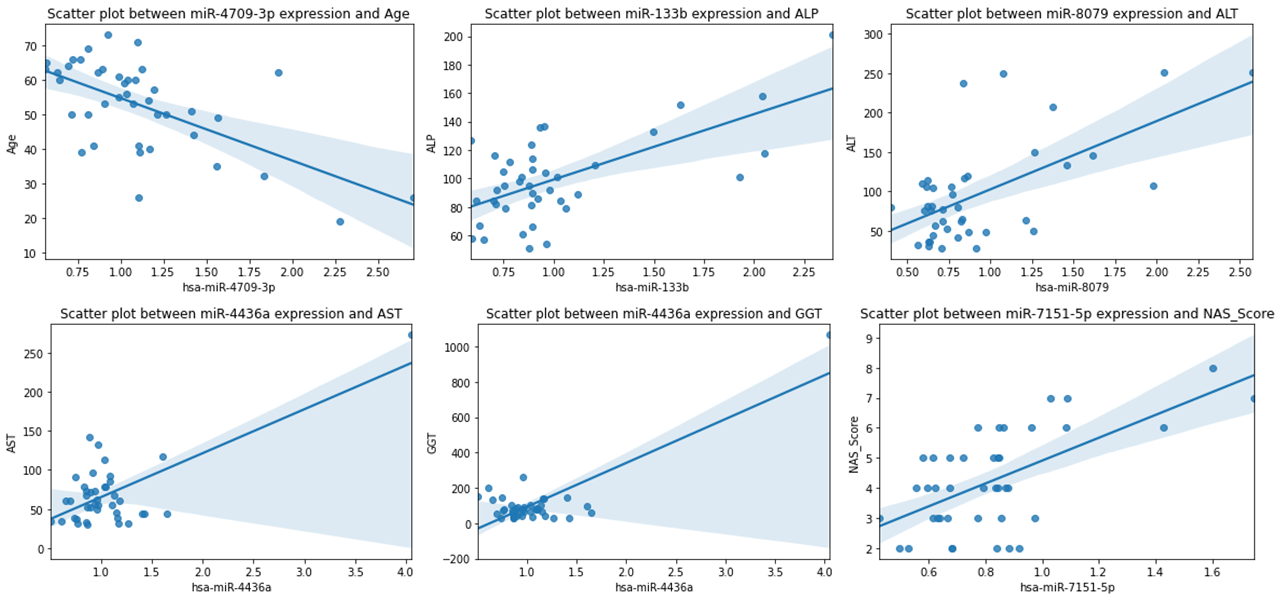

Supplement: S5 Fig — In the scatter plot, the x-axis indicates the miRNA expression levels, and the y-axis indicates each clinical parameter. The lines and shades indicate the regression and confidence intervals, respectively. (TIF) [file pone.0255822.s005.tif]

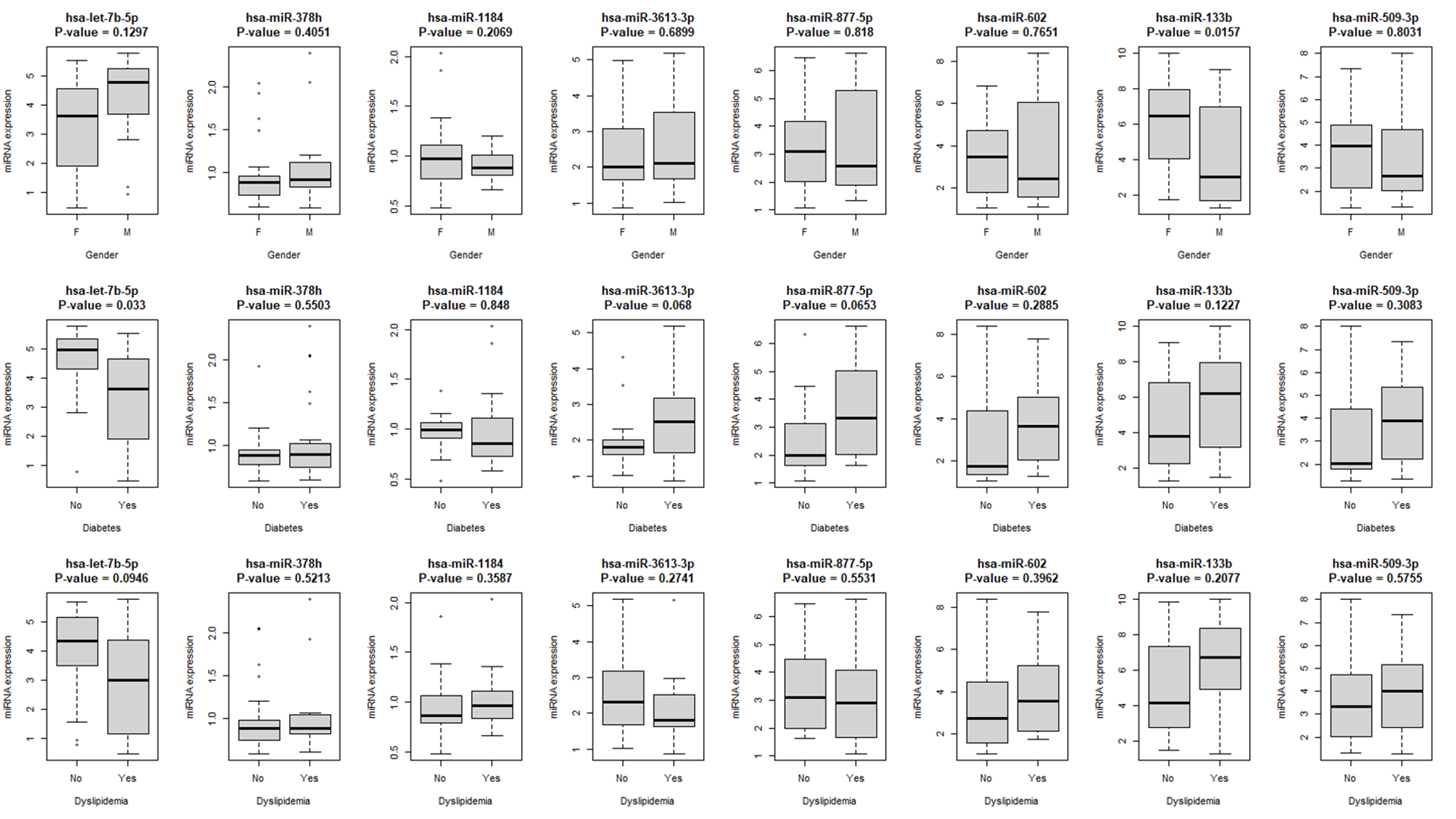

Supplement: S6 Fig — Eight miRNAs (let-7b-5p, miR-378h, -1184, -3613-3p, -877-5p, -602, -133b, and 509-3p) and three confounding variables (sex, diabetes, and dyslipidemia) were listed, and the expression levels are presented as boxplots with significance values (p-values). NAFLD, nonalcoholic fatty liver disease. (TIF) [file pone.0255822.s006.tif]
