# Supplementary material for: Evaluation of the severity of nonalcoholic fatty liver disease through analysis of serum exosomal miRNA expression
Source: PLoS One. 2021 Aug 6;16(8):e0255822. doi: 10.1371/journal.pone.0255822 (PMC8345824; doi:10.1371/journal.pone.0255822)
Supplement: S2 Table — Each correlated miRNA with each variable is listed, and its genomic location is provided (miRbase Release 20). Correlations between variables and miRNA expression levels were estimated using the “pingouin” library in Python. Samples with “power” > 0.9, were selected. “n,” “r,” “CI95,” “adj_r2,” “p-val,” and “BF10” represent “sample number,” “correlation coefficient,” “95% confidence interval,” “adjusted r square,” “p-value,” and “Bayes factor of the alternative hypothesis,” respectively. Power was calculated using the “1-type II error”. (DOCX) [file pone.0255822.s008.docx]

**S1 Table.** **Correlations between the 147 expression patterns of 117 miRNAs and the 15 selected variables.** Each correlated miRNA with each variable is listed, and its genomic location is provided (miRbase Release 20). Correlations between variables and miRNA expression levels were estimated in Python using the “Pingouin” library. Samples with “power” > 0.9 were selected. “n,” “r,” “CI95,” “adj_r2,” “p-val,” and “BF10” stand for “sample number,” “correlation coefficient,” “95% confidence interval,” “adjusted r square,” “*p*-value,” and “Bayes factor of the alternative hypothesis,” respectively. Power was calculated using the “1-type II error.”

| Variables | Name | Locations | n | r | CI95 | r2 | adj_r2 | p_val | BF10 | power |
| --- | --- | --- | --- | --- | --- | --- | --- | --- | --- | --- |
| Age | hsa-miR-4709-3p | chr7:21510723-21510749 (+) | 41 | -0.620833999 | [-0.78 -0.39] | 0.385434854 | 0.35308932 | 1.49E-05 | 1654.448 | 0.994799348 |
|  | hsa-miR-4999-5p | chr19:10662798-10662820 (+) | 41 | -0.603908422 | [-0.77 -0.36] | 0.364705382 | 0.331268823 | 2.91E-05 | 893.532 | 0.991665559 |
|  | hsa-miR-3064-5p | chr12:9392068-9392093 (-) // chr12:12264940-12264965 (+) // chr2:232578078-232578103 (+) | 41 | -0.573005966 | [-0.75 -0.32] | 0.328335837 | 0.292985092 | 9.02E-05 | 317.742 | 0.982137162 |
|  | hsa-miR-6754-5p | chr1:167967937-167967958 (+) | 41 | -0.56432271 | [-0.74 -0.31] | 0.318460121 | 0.282589601 | 0.000121564 | 242.284 | 0.978321538 |
|  | hsa-miR-32-3p | chr1:167967903-167967924 (+) // chr4:36428027-36428048 (-) | 41 | -0.559887598 | [-0.74 -0.3 ] | 0.313474123 | 0.277341182 | 0.000141101 | 211.603 | 0.976145135 |
|  | hsa-miR-6883-5p | chr1:21314870-21314891 (-) | 41 | -0.552094568 | [-0.73 -0.29] | 0.304808412 | 0.268219381 | 0.000182419 | 167.619 | 0.971921067 |
|  | hsa-miR-6772-3p | chr9:69002239-69002260 (-) | 41 | -0.551489418 | [-0.73 -0.29] | 0.304140578 | 0.267516398 | 0.000186044 | 164.656 | 0.971570849 |
|  | hsa-miR-1183 | chr22:20073635-20073652 (+) | 41 | -0.537692545 | [-0.73 -0.28] | 0.289113272 | 0.251698181 | 0.000288476 | 110.713 | 0.962659572 |
|  | hsa-miR-483-5p | chr22:22007605-22007625 (+) | 41 | -0.530346877 | [-0.72 -0.27] | 0.28126781 | 0.2434398 | 0.000361598 | 90.29 | 0.957147786 |
|  | hsa-miR-548b-5p | chr22:22007605-22007625 (+) | 41 | -0.528399642 | [-0.72 -0.26] | 0.279206182 | 0.241269665 | 0.000383585 | 85.61 | 0.955592173 |
|  | hsa-miR-1299 | chr18:19405673-19405694 (-) // chr20:61162177-61162198 (+) | 41 | -0.523593775 | [-0.72 -0.26] | 0.274150441 | 0.235947833 | 0.000443072 | 75.184 | 0.951578056 |
|  | hsa-miR-6831-5p | chr6:52013786-52013807 (+) | 41 | -0.519155462 | [-0.71 -0.25] | 0.269522393 | 0.231076204 | 0.000505218 | 66.81 | 0.947644935 |
|  | hsa-miR-4538 | chr3:52328248-52328269 (-) | 41 | -0.509476128 | [-0.71 -0.24] | 0.259565925 | 0.220595711 | 0.000668552 | 51.951 | 0.938288089 |
|  | hsa-miR-1306-3p | chr5:148808541-148808561 (+) | 41 | -0.499857307 | [-0.7 -0.23] | 0.249857327 | 0.210376134 | 0.000876029 | 40.782 | 0.927890791 |
|  | hsa-miR-6769a-5p | chr17:27188566-27188585 (-) | 41 | -0.48898464 | [-0.69 -0.21] | 0.239105978 | 0.199058924 | 0.001177991 | 31.306 | 0.914767594 |
|  | hsa-miR-598-5p | chr17:27188601-27188622 (-) | 41 | -0.486920276 | [-0.69 -0.21] | 0.237091355 | 0.196938268 | 0.001244772 | 29.805 | 0.912107938 |
|  | hsa-miR-4296 | chr19:13985577-13985598 (+) | 41 | -0.485529649 | [-0.69 -0.21] | 0.23573904 | 0.195514779 | 0.001291629 | 28.84 | 0.910285741 |
|  | hsa-miR-1238-5p | chr13:92003193-92003215 (+) | 41 | -0.48214107 | [-0.69 -0.2 ] | 0.232460011 | 0.19206317 | 0.001412417 | 26.634 | 0.905742093 |
|  | hsa-miR-493-3p | chr13:92003193-92003215 (+) | 41 | -0.479725363 | [-0.69 -0.2 ] | 0.230136424 | 0.189617288 | 0.001504529 | 25.179 | 0.902413025 |
| Albumin | hsa-miR-6756-3p | chr13:92003461-92003483 (+) | 41 | -0.500139205 | [-0.7 -0.23] | 0.250139224 | 0.210672868 | 0.000869214 | 41.068 | 0.928211496 |
|  | hsa-miR-6749-3p | chr8:113655782-113655804 (+) | 41 | -0.491757093 | [-0.69 -0.22] | 0.241825038 | 0.201921093 | 0.001093313 | 33.459 | 0.918254608 |
|  | hsa-miR-487b-3p | chr8:113655782-113655804 (+) | 41 | -0.486192132 | [-0.69 -0.21] | 0.236382789 | 0.19619241 | 0.001269115 | 29.295 | 0.911156894 |
|  | hsa-miR-628-3p | chr17:1953584-1953604 (-) | 41 | -0.480404122 | [-0.69 -0.2 ] | 0.230788121 | 0.190303285 | 0.001478125 | 25.579 | 0.903355998 |
|  | hsa-miR-590-5p | chr17:1953584-1953604 (-) | 41 | 0.479345447 | [0.2 0.69] | 0.229772058 | 0.189233745 | 0.001519489 | 24.959 | 0.901882635 |
|  | hsa-miR-3681-5p | chr20:57392681-57392702 (-) | 41 | 0.488152499 | [0.21 0.69] | 0.238292862 | 0.198203013 | 0.001204518 | 30.69 | 0.913702003 |
|  | hsa-miR-5705 | chr14:101507751-101507772 (+) | 41 | 0.488401347 | [0.21 0.69] | 0.238535876 | 0.198458817 | 0.00119653 | 30.873 | 0.914021584 |
| ALP | hsa-miR-3615 | chr17:62496935-62496955 (-) | 41 | 0.485174385 | [0.21 0.69] | 0.235394184 | 0.195151773 | 0.001303848 | 28.599 | 0.909816269 |
|  | hsa-miR-3168 | chr8:135812813-135812834 (-) | 41 | 0.488936162 | [0.21 0.69] | 0.239058571 | 0.199009022 | 0.001179522 | 31.27 | 0.914705758 |
|  | hsa-miR-518f-5p | chr1:67094135-67094154 (+) | 41 | 0.492921718 | [0.22 0.7 ] | 0.24297182 | 0.203128231 | 0.001059386 | 34.414 | 0.919690496 |
|  | hsa-miR-4294 | chr10:97824126-97824147 (-) | 41 | 0.497642051 | [0.22 0.7 ] | 0.247647611 | 0.208050117 | 0.000931257 | 38.613 | 0.925336495 |
|  | hsa-miR-1255b-5p | chr10:97824126-97824147 (-) | 41 | 0.504662696 | [0.23 0.7 ] | 0.254684436 | 0.215457301 | 0.000766135 | 45.98 | 0.933224841 |
|  | hsa-miR-644a | chr13:41675212-41675228 (-) | 41 | 0.506471165 | [0.24 0.7 ] | 0.256513041 | 0.217382149 | 0.000728075 | 48.127 | 0.935159722 |
|  | hsa-miR-144-3p | chr14:95604260-95604281 (-) | 41 | 0.507971992 | [0.24 0.71] | 0.258035544 | 0.218984784 | 0.00069778 | 49.996 | 0.936735643 |
|  | hsa-miR-450a-1-3p | chrX:153246556-153246577 (+) // chrX:153246599-153246620 (-) | 41 | 0.527108482 | [0.26 0.72] | 0.277843351 | 0.239835107 | 0.000398818 | 82.658 | 0.954538332 |
|  | hsa-miR-4778-5p | chr9:111808511-111808532 (-) | 41 | 0.539769783 | [0.28 0.73] | 0.291351419 | 0.254054125 | 0.000270367 | 117.392 | 0.964118647 |
|  | hsa-miR-596 | chr17:17717171-17717192 (-) | 41 | 0.55662555 | [0.3 0.74] | 0.309832003 | 0.273507372 | 0.000157237 | 191.794 | 0.974440566 |
|  | hsa-miR-380-3p | chr17:17717171-17717192 (-) | 41 | 0.568661686 | [0.32 0.75] | 0.323376113 | 0.28776433 | 0.000104853 | 277.157 | 0.980299624 |
|  | hsa-miR-6746-3p | chr17:72744802-72744822 (+) | 41 | 0.574139005 | [0.32 0.75] | 0.329635597 | 0.29435326 | 8.67E-05 | 329.382 | 0.982593729 |
|  | hsa-miR-133b | chr7:132719620-132719638 (-) | 41 | 0.639943211 | [0.41 0.79] | 0.409527313 | 0.378449803 | 6.63E-06 | 3476.493 | 0.997102679 |
| ALT | hsa-miR-19b-1-5p | chr7:132719620-132719638 (-) | 41 | -0.478590422 | [-0.69 -0.2 ] | 0.229048792 | 0.188472412 | 0.001549611 | 24.527 | 0.900823044 |
|  | hsa-miR-6515-5p | chr5:89312458-89312478 (-) | 41 | 0.493628461 | [0.22 0.7 ] | 0.243669057 | 0.203862166 | 0.001039257 | 35.008 | 0.920553549 |
|  | hsa-miR-4252 | chr5:89312458-89312478 (-) | 41 | 0.504442522 | [0.23 0.7 ] | 0.254462258 | 0.21522343 | 0.000770888 | 45.726 | 0.932986584 |
|  | hsa-miR-569 | chr8:1749299-1749320 (+) | 41 | 0.534035535 | [0.27 0.72] | 0.285193953 | 0.247572582 | 0.000323026 | 99.962 | 0.959985111 |
|  | hsa-miR-8079 | chr2:12339264-12339285 (+) | 41 | 0.621373349 | [0.39 0.78] | 0.386104839 | 0.353794567 | 1.45E-05 | 1688.3 | 0.994880462 |
| AST | hsa-miR-1256 | chrX:1412820-1412842 (+) // chrY:1362820-1362842 (+) | 41 | 0.488497502 | [0.21 0.69] | 0.238629809 | 0.198557694 | 0.001193456 | 30.944 | 0.914144859 |
|  | hsa-miR-5581-5p | chr19:54290932-54290954 (-) | 41 | 0.489736126 | [0.21 0.69] | 0.239841473 | 0.19983313 | 0.001154482 | 31.873 | 0.915722363 |
|  | hsa-miR-4705 | chr14:101491393-101491414 (+) | 41 | 0.494449667 | [0.22 0.7 ] | 0.244480474 | 0.204716288 | 0.001016295 | 35.713 | 0.921548521 |
|  | hsa-miR-300 | chr14:101520653-101520674 (+) | 41 | 0.496903374 | [0.22 0.7 ] | 0.246912963 | 0.207276803 | 0.000950349 | 37.919 | 0.924471274 |
|  | hsa-miR-3157-5p | chr5:170813682-170813702 (-) | 41 | 0.497514746 | [0.22 0.7 ] | 0.247520922 | 0.20791676 | 0.000934523 | 38.492 | 0.925187863 |
|  | hsa-miR-212-3p | chr6:36590257-36590278 (-) | 41 | 0.504283638 | [0.23 0.7 ] | 0.254301988 | 0.215054724 | 0.000774334 | 45.544 | 0.932814287 |
|  | hsa-miR-4694-3p | chr8:12584783-12584803 (-) // chr8:12584751-12584771 (+) | 41 | 0.518009185 | [0.25 0.71] | 0.268333515 | 0.229824753 | 0.000522487 | 64.821 | 0.946593168 |
|  | hsa-miR-4512 | chr6:33665968-33665989 (+) | 41 | 0.518752245 | [0.25 0.71] | 0.269103891 | 0.230635675 | 0.000511234 | 66.103 | 0.947276661 |
|  | hsa-miR-3660 | chr1:6489904-6489922 (-) | 41 | 0.52270161 | [0.26 0.72] | 0.273216973 | 0.234965234 | 0.000454984 | 73.41 | 0.950805063 |
|  | hsa-miR-599 | chr5:86410741-86410761 (-) | 41 | 0.524276884 | [0.26 0.72] | 0.274866251 | 0.236701317 | 0.000434141 | 76.575 | 0.952163984 |
|  | hsa-miR-3654 | chr9:96581649-96581664 (+) | 41 | 0.526897248 | [0.26 0.72] | 0.27762071 | 0.239600748 | 0.000401361 | 82.186 | 0.954364215 |
|  | hsa-miR-3912-3p | chr9:96581649-96581664 (+) | 41 | 0.539389739 | [0.28 0.73] | 0.29094129 | 0.253622411 | 0.000273602 | 116.137 | 0.963854909 |
|  | hsa-miR-130b-5p | chr10:50193606-50193622 (-) | 41 | 0.552913106 | [0.3 0.74] | 0.305712903 | 0.269171477 | 0.000177616 | 171.723 | 0.972389578 |
|  | hsa-miR-518f-5p | chr10:126721413-126721429 (-) | 41 | 0.559704447 | [0.3 0.74] | 0.313269068 | 0.277125335 | 0.000141966 | 210.432 | 0.976051797 |
|  | hsa-miR-4427 | chr1:233759941-233759961 (+) | 41 | 0.563363157 | [0.31 0.74] | 0.317378047 | 0.281450575 | 0.00012557 | 235.249 | 0.977864147 |
|  | hsa-miR-33b-3p | chr1:233759941-233759961 (+) | 41 | 0.57304376 | [0.32 0.75] | 0.32837915 | 0.293030685 | 9.01E-05 | 318.123 | 0.98215254 |
|  | hsa-miR-569 | chr2:89111939-89111959 (+) | 41 | 0.577399479 | [0.33 0.75] | 0.333390158 | 0.29830543 | 7.74E-05 | 365.601 | 0.983856919 |
|  | hsa-miR-4650-5p | chr2:89111939-89111959 (+) | 41 | 0.583666079 | [0.34 0.76] | 0.340666092 | 0.305964307 | 6.19E-05 | 448.265 | 0.986082369 |
|  | hsa-miR-2053 | chr2:182170320-182170340 (-) | 41 | 0.586504649 | [0.34 0.76] | 0.343987703 | 0.30946074 | 5.58E-05 | 492.351 | 0.987007143 |
|  | hsa-miR-5682 | chr5:535976-535992 (-) | 41 | 0.588487147 | [0.34 0.76] | 0.346317123 | 0.311912761 | 5.19E-05 | 525.978 | 0.987623643 |
|  | hsa-miR-548ah-3p | chrX:133674559-133674580 (-) | 41 | 0.618306634 | [0.38 0.78] | 0.382303094 | 0.34979273 | 1.65E-05 | 1505.434 | 0.994405074 |
|  | hsa-miR-644a | chr15:66789303-66789324 (-) | 41 | 0.625309424 | [0.39 0.78] | 0.391011875 | 0.358959869 | 1.24E-05 | 1959.606 | 0.995441494 |
|  | hsa-miR-19a-3p | chr15:66789303-66789324 (-) | 41 | 0.631902766 | [0.4 0.79] | 0.399301106 | 0.367685375 | 9.38E-06 | 2527.469 | 0.996267336 |
|  | hsa-miR-577 | chr14:106324390-106324411 (-) | 41 | 0.638002025 | [0.41 0.79] | 0.407046584 | 0.375838509 | 7.22E-06 | 3216.146 | 0.996916885 |
|  | hsa-miR-559 | chr7:66579352-66579370 (-) // chr7:72162888-72162906 (+) | 41 | 0.654208719 | [0.43 0.8 ] | 0.427989048 | 0.397883208 | 3.50E-06 | 6269.95 | 0.998201961 |
|  | hsa-miR-4291 | chr7:66579352-66579370 (-) // chr7:72162888-72162906 (+) | 41 | 0.669382999 | [0.46 0.81] | 0.448073599 | 0.419024841 | 1.71E-06 | 12170 | 0.998962233 |
|  | hsa-miR-4436a | chr9:137271295-137271316 (+) | 41 | 0.702547834 | [0.5 0.83] | 0.493573459 | 0.466919431 | 3.05E-07 | 60110 | 0.999738026 |
| Bilirubin | hsa-miR-5585-3p | chr10:112657889-112657909 (+) | 41 | 0.514927187 | [0.25 0.71] | 0.265150008 | 0.226473692 | 0.000571567 | 59.798 | 0.943690924 |
| BUN | hsa-miR-4791 | chr11:19781559-19781579 (-) | 41 | -0.535397113 | [-0.72 -0.27] | 0.286650069 | 0.249105336 | 0.00030975 | 103.822 | 0.960996793 |
|  | hsa-miR-5000-3p | chr11:19781559-19781579 (-) | 41 | -0.506735878 | [-0.7 -0.24] | 0.25678125 | 0.217664474 | 0.000722648 | 48.451 | 0.935439638 |
|  | hsa-miR-4437 | chr13:102698324-102698345 (-) | 41 | -0.485825951 | [-0.69 -0.21] | 0.236026855 | 0.195817742 | 0.001281516 | 29.042 | 0.910676065 |
|  | hsa-miR-548aw | chr13:102698324-102698345 (-) | 41 | -0.482064173 | [-0.69 -0.2 ] | 0.232385867 | 0.191985123 | 0.00141527 | 26.586 | 0.905637277 |
|  | hsa-miR-891a-5p | chr14:74946838-74946860 (-) | 41 | 0.489522358 | [0.21 0.69] | 0.239632139 | 0.199612778 | 0.001161127 | 31.711 | 0.915451497 |
|  | hsa-miR-181c-3p | chr14:74946838-74946860 (-) | 41 | 0.523358229 | [0.26 0.72] | 0.273903835 | 0.235688248 | 0.000446189 | 74.711 | 0.951374828 |
| Cr | hsa-miR-3117-5p | chr19:50436330-50436353 (+) | 41 | 0.486572938 | [0.21 0.69] | 0.236753224 | 0.196582341 | 0.001256332 | 29.56 | 0.911655115 |
|  | hsa-miR-3926 | chr2:19548200-19548222 (+) | 41 | 0.499576462 | [0.23 0.7 ] | 0.249576642 | 0.210080676 | 0.000882865 | 40.5 | 0.927570313 |
| CRP | hsa-miR-1244 | chr2:66585429-66585450 (-) | 41 | -0.570304154 | [-0.75 -0.32] | 0.325246828 | 0.289733503 | 9.91E-05 | 291.783 | 0.981010807 |
|  | hsa-miR-562 | chr3:19356399-19356416 (-) | 41 | -0.505429002 | [-0.7 -0.23] | 0.255458476 | 0.21627208 | 0.000749797 | 46.876 | 0.934049521 |
|  | hsa-miR-4456 | chr11:2155411-2155432 (-) | 41 | 0.495229819 | [0.22 0.7 ] | 0.245252574 | 0.205529025 | 0.0009949 | 36.398 | 0.922485941 |
|  | hsa-miR-6830-3p | chr14:101512842-101512863 (+) | 41 | 0.498117855 | [0.22 0.7 ] | 0.248121398 | 0.20854884 | 0.00091914 | 39.068 | 0.92589023 |
| GGT | hsa-miR-3934-3p | chr1:176998548-176998568 (-) | 41 | -0.501465405 | [-0.7 -0.23] | 0.251467552 | 0.212071107 | 0.000837785 | 42.444 | 0.92970718 |
|  | hsa-miR-4705 | chr1:176998548-176998568 (-) | 41 | 0.48134974 | [0.2 0.69] | 0.231697572 | 0.191260603 | 0.001442025 | 26.147 | 0.904659827 |
|  | hsa-miR-8074 | chr14:101335453-101335474 (+) | 41 | 0.481475725 | [0.2 0.69] | 0.231818874 | 0.191388288 | 0.001437275 | 26.224 | 0.904832669 |
|  | hsa-miR-4757-5p | chr19:8454224-8454244 (-) | 41 | 0.487748753 | [0.21 0.69] | 0.237898846 | 0.197788259 | 0.001217578 | 30.397 | 0.913181822 |
|  | hsa-miR-5588-5p | chr2:75318000-75318021 (+) | 41 | 0.492017211 | [0.22 0.69] | 0.242080936 | 0.202190459 | 0.001085653 | 33.67 | 0.918576792 |
|  | hsa-miR-548c-3p | chrX:49775290-49775307 (+) | 41 | 0.492773228 | [0.22 0.7 ] | 0.242825454 | 0.202974162 | 0.001063659 | 34.29 | 0.919508369 |
|  | hsa-miR-1255b-2-3p | chrX:146280575-146280596 (-) | 41 | 0.504528424 | [0.23 0.7 ] | 0.254548931 | 0.215314664 | 0.000769031 | 45.825 | 0.933079611 |
|  | hsa-miR-6795-3p | chr19:54203321-54203341 (+) | 41 | 0.509028731 | [0.24 0.71] | 0.259110249 | 0.220116052 | 0.000677129 | 51.361 | 0.937829132 |
|  | hsa-miR-5682 | chr19:54203284-54203305 (+) | 41 | 0.509109314 | [0.24 0.71] | 0.259192293 | 0.220202414 | 0.000675577 | 51.466 | 0.937911972 |
|  | hsa-miR-144-5p | chr19:54203284-54203305 (+) | 41 | 0.510674875 | [0.24 0.71] | 0.260788828 | 0.221882976 | 0.000646046 | 53.572 | 0.939506132 |
|  | hsa-miR-371b-3p | chr19:54203284-54203305 (+) | 41 | 0.515048683 | [0.25 0.71] | 0.265275146 | 0.226605417 | 0.000569557 | 59.987 | 0.943807401 |
|  | hsa-miR-4669 | chr4:77496750-77496771 (+) | 41 | 0.524078842 | [0.26 0.72] | 0.274658632 | 0.236482771 | 0.000436714 | 76.169 | 0.951994644 |
|  | hsa-miR-7151-5p | chr4:77496750-77496771 (+) | 41 | 0.532623383 | [0.27 0.72] | 0.283687668 | 0.245987019 | 0.000337332 | 96.128 | 0.958915651 |
|  | hsa-miR-3173-3p | chr9:135821101-135821120 (+) | 41 | 0.540150819 | [0.28 0.73] | 0.291762908 | 0.254487271 | 0.000267159 | 118.666 | 0.964381639 |
|  | hsa-miR-4680-3p | chr6:119390263-119390284 (-) | 41 | 0.541852572 | [0.28 0.73] | 0.29360421 | 0.256425484 | 0.000253244 | 124.546 | 0.965538771 |
|  | hsa-miR-513b-3p | chr12:65016349-65016370 (+) | 41 | 0.547967799 | [0.29 0.73] | 0.300268709 | 0.263440747 | 0.000208462 | 148.529 | 0.969466963 |
|  | hsa-miR-6079 | chr1:37966574-37966595 (-) | 41 | 0.553310825 | [0.3 0.74] | 0.306152869 | 0.269634599 | 0.000175324 | 173.757 | 0.972615074 |
|  | hsa-miR-518f-3p | chr1:32552587-32552608 (+) | 41 | 0.560733238 | [0.31 0.74] | 0.314421764 | 0.278338699 | 0.00013717 | 217.102 | 0.976572488 |
|  | hsa-miR-3654 | chr3:184971040-184971060 (-) | 41 | 0.56191338 | [0.31 0.74] | 0.315746646 | 0.279733312 | 0.00013185 | 225.045 | 0.977159056 |
|  | hsa-miR-6072 | chr2:47604829-47604849 (+) | 41 | 0.562142782 | [0.31 0.74] | 0.316004507 | 0.280004744 | 0.000130837 | 226.626 | 0.977271757 |
|  | hsa-miR-4427 | chr2:47604829-47604849 (+) | 41 | 0.579767938 | [0.33 0.75] | 0.336130862 | 0.301190381 | 7.11E-05 | 394.676 | 0.984728618 |
|  | hsa-miR-559 | chr2:233037423-233037442 (+) | 41 | 0.581510693 | [0.33 0.75] | 0.338154686 | 0.303320723 | 6.68E-05 | 417.704 | 0.985346081 |
|  | hsa-miR-212-3p | chr3:120768531-120768552 (+) | 41 | 0.584323572 | [0.34 0.76] | 0.341434037 | 0.30677267 | 6.04E-05 | 458.074 | 0.98630106 |
|  | hsa-miR-6503-3p | chr3:120768531-120768552 (+) | 41 | 0.585742119 | [0.34 0.76] | 0.34309383 | 0.308519821 | 5.74E-05 | 480.055 | 0.986763643 |
|  | hsa-miR-3202 | chr3:170824468-170824488 (-) | 41 | 0.593935868 | [0.35 0.76] | 0.352759815 | 0.318694542 | 4.25E-05 | 632.208 | 0.989199099 |
|  | hsa-miR-488-5p | chr3:170824468-170824488 (-) | 41 | 0.603955848 | [0.36 0.77] | 0.364762667 | 0.331329123 | 2.90E-05 | 895.029 | 0.991676093 |
|  | hsa-miR-659-5p | chr4:88221657-88221679 (-) | 41 | 0.604569586 | [0.36 0.77] | 0.365504385 | 0.332109879 | 2.84E-05 | 914.66 | 0.99181145 |
|  | hsa-miR-4512 | chr8:81153633-81153652 (+) | 41 | 0.605697984 | [0.37 0.77] | 0.366870047 | 0.333547418 | 2.71E-05 | 952.002 | 0.992055671 |
|  | hsa-miR-3690 | chr4:115577930-115577950 (+) | 41 | 0.622330835 | [0.39 0.78] | 0.387295668 | 0.355048072 | 1.40E-05 | 1750.282 | 0.995021899 |
|  | hsa-miR-599 | chr4:115577930-115577950 (+) | 41 | 0.638964648 | [0.41 0.79] | 0.408275821 | 0.377132444 | 6.92E-06 | 3342.483 | 0.997010223 |
|  | hsa-miR-3925-5p | chr7:73605543-73605564 (+) | 41 | 0.639952219 | [0.41 0.79] | 0.409538843 | 0.37846194 | 6.63E-06 | 3477.754 | 0.997103519 |
|  | hsa-miR-19a-3p | chr8:1765412-1765432 (+) | 41 | 0.645200124 | [0.42 0.8 ] | 0.4162832 | 0.385561263 | 5.26E-06 | 4304.559 | 0.997559492 |
|  | hsa-miR-577 | chr8:1765412-1765432 (+) | 41 | 0.649725761 | [0.43 0.8 ] | 0.422143564 | 0.391730067 | 4.29E-06 | 5191.441 | 0.997902851 |
|  | hsa-miR-4650-5p | chr8:10892767-10892789 (-) | 41 | 0.656712978 | [0.44 0.8 ] | 0.431271935 | 0.401338879 | 3.12E-06 | 6977.017 | 0.998352767 |
|  | hsa-miR-4694-3p | chr8:100548879-100548898 (-) | 41 | 0.65956438 | [0.44 0.8 ] | 0.435025171 | 0.405289654 | 2.73E-06 | 7889.554 | 0.998511316 |
|  | hsa-miR-3157-5p | chr8:100548879-100548898 (-) | 41 | 0.660421615 | [0.44 0.8 ] | 0.436156709 | 0.406480746 | 2.62E-06 | 8188.716 | 0.998556374 |
|  | hsa-miR-130b-5p | chr10:2118225-2118246 (-) | 41 | 0.661416562 | [0.44 0.81] | 0.437471868 | 0.407865125 | 2.50E-06 | 8551.51 | 0.998607215 |
|  | hsa-miR-518f-5p | chr10:2118225-2118246 (-) | 41 | 0.682083764 | [0.47 0.82] | 0.465238261 | 0.437092906 | 9.07E-07 | 21880 | 0.999368558 |
|  | hsa-miR-3660 | chr1:44304329-44304352 (+) | 41 | 0.682752202 | [0.47 0.82] | 0.466150569 | 0.438053231 | 8.76E-07 | 22590 | 0.999385465 |
|  | hsa-miR-548ah-3p | chr15:55665152-55665172 (-) | 41 | 0.713558377 | [0.52 0.84] | 0.509165557 | 0.483332165 | 1.64E-07 | 107400 | 0.999844031 |
|  | hsa-miR-4291 | chr20:33054190-33054208 (+) | 41 | 0.72201771 | [0.53 0.84] | 0.521309574 | 0.496115341 | 9.93E-08 | 170900 | 0.999897756 |
|  | hsa-miR-2053 | chr20:33054190-33054208 (+) | 41 | 0.723876938 | [0.54 0.84] | 0.523997822 | 0.498945076 | 8.88E-08 | 189800 | 0.999907092 |
|  | hsa-miR-644a | chr20:33054190-33054208 (+) | 41 | 0.742646561 | [0.56 0.85] | 0.551523915 | 0.52791991 | 2.71E-08 | 573100 | 0.999966874 |
|  | hsa-miR-33b-3p | chr11:59976557-59976577 (-) | 41 | 0.764716943 | [0.6 0.87] | 0.584792003 | 0.562938951 | 5.88E-09 | 2394000 | 0.999991696 |
|  | hsa-miR-4436a | chr19:13051300-13051320 (+) | 41 | 0.828842343 | [0.7 0.91] | 0.68697963 | 0.670504874 | 2.21E-11 | 451100000 | 0.999999967 |
| Hb | hsa-miR-4751 | chr22:38243739-38243760 (-) | 41 | -0.520059306 | [-0.71 -0.25] | 0.270461682 | 0.232064929 | 0.000491964 | 68.426 | 0.94846378 |
|  | hsa-miR-30b-5p | chr11:61645688-61645709 (-) | 41 | -0.506832978 | [-0.7 -0.24] | 0.256879668 | 0.217768071 | 0.000720666 | 48.57 | 0.935542105 |
|  | hsa-miR-296-3p | chr11:64669859-64669879 (-) | 41 | 0.494069151 | [0.22 0.7 ] | 0.244104326 | 0.204320344 | 0.001026878 | 35.385 | 0.921088538 |
|  | hsa-miR-4709-3p | chr11:71184554-71184575 (+) | 41 | 0.503211525 | [0.23 0.7 ] | 0.253221839 | 0.213917725 | 0.000797948 | 44.335 | 0.931643664 |
| NAS_Score | hsa-miR-7157-5p | chr11:119183660-119183679 (-) | 41 | 0.489304777 | [0.21 0.69] | 0.239419164 | 0.199388594 | 0.001167925 | 31.546 | 0.915175205 |
|  | hsa-miR-596 | chr16:4721324-4721344 (+) | 41 | 0.500899867 | [0.23 0.7 ] | 0.250900677 | 0.211474397 | 0.000851061 | 41.851 | 0.929072005 |
|  | hsa-miR-3674 | chr16:57806202-57806224 (-) | 41 | 0.516686131 | [0.25 0.71] | 0.266964558 | 0.228383746 | 0.000543074 | 62.609 | 0.945360615 |
|  | hsa-miR-6072 | chr19:15290094-15290114 (-) | 41 | 0.530696927 | [0.27 0.72] | 0.281639229 | 0.243830767 | 0.000357768 | 91.161 | 0.957423169 |
|  | hsa-miR-7151-5p | chr3:127294148-127294169 (-) | 41 | 0.627189352 | [0.4 0.78] | 0.393366483 | 0.361438403 | 1.14E-05 | 2105.759 | 0.995690984 |
| PLT | hsa-miR-4280 | chr5:131553542-131553564 (-) | 41 | -0.48137743 | [-0.69 -0.2 ] | 0.23172423 | 0.191288663 | 0.00144098 | 26.164 | 0.904697833 |
|  | hsa-miR-143-3p | chr5:139943312-139943335 (-) | 41 | 0.494702076 | [0.22 0.7 ] | 0.244730144 | 0.204979099 | 0.001009328 | 35.933 | 0.921852643 |
|  | hsa-miR-135a-3p | chr17:8048363-8048384 (-) | 41 | 0.502159702 | [0.23 0.7 ] | 0.252164366 | 0.212804596 | 0.000821738 | 43.184 | 0.930481622 |
| PT_INR | hsa-miR-488-5p | chr10:69163146-69163168 (-) | 41 | -0.542043919 | [-0.73 -0.28] | 0.29381161 | 0.2566438 | 0.000251721 | 125.227 | 0.96566711 |
|  | hsa-miR-6825-5p | chr10:69163146-69163168 (-) | 41 | -0.519451642 | [-0.71 -0.25] | 0.269830008 | 0.231400008 | 0.00050084 | 67.335 | 0.947914276 |
|  | hsa-miR-133a-3p | chr2:141344232-141344254 (-) | 41 | -0.494515427 | [-0.7 -0.22] | 0.244545507 | 0.204784745 | 0.001014476 | 35.771 | 0.92162783 |
|  | hsa-miR-5708 | chr19:51710195-51710218 (-) | 41 | 0.485908509 | [0.21 0.69] | 0.236107079 | 0.195902189 | 0.001278711 | 29.099 | 0.91078462 |
|  | hsa-miR-500b-5p | chr13:44770307-44770326 (-) | 41 | 0.489238931 | [0.21 0.69] | 0.239354732 | 0.199320771 | 0.001169989 | 31.497 | 0.915091474 |
| WBC | hsa-miR-382-5p | chrX:145109359-145109380 (-) | 41 | 0.48733328 | [0.21 0.69] | 0.237493725 | 0.197361816 | 0.001231149 | 30.098 | 0.912644371 |
